# Thermal stability and reduction of iron oxide nanowires at moderate temperatures

**DOI:** 10.3762/bjnano.5.36

**Published:** 2014-03-19

**Authors:** Annalisa Paolone, Marco Angelucci, Stefania Panero, Maria Grazia Betti, Carlo Mariani

**Affiliations:** 1 CNR-ISC, U.O.S. La Sapienza, Piazzale Aldo Moro 2, I - 00185 Roma, Italy; 2 Dipartimento di Fisica, Università di Roma La Sapienza, Piazzale Aldo Moro 2, I - 00185 Roma, Italy; 3 Dipartimento di Chimica, Università di Roma La Sapienza, Piazzale Aldo Moro 2, I - 00185 Roma, Italy

**Keywords:** IR spectroscopy, iron oxide, nanowires, scanning electron microscopy (SEM), thermogravimetry, XPS

## Abstract

**Background:** The thermal stability of iron oxide nanowires, which were obtained with a hard template method and are promising elements of Li-ion based batteries, has been investigated by means of thermogravimetry, infrared and photoemission spectroscopy measurements.

**Results:** The chemical state of the nanowires is typical of the Fe_2_O_3_ phase and the stoichiometry changes towards a Fe_3_O_4_ phase by annealing above 440 K. The shape and morphology of the nanowires is not modified by moderate thermal treatment, as imaged by scanning electron microscopy.

**Conclusion:** This complementary spectroscopy–microscopy study allows to assess the temperature limits of these Fe_2_O_3_ nanowires during operation, malfunctioning or abuse in advanced Li-ion based batteries.

## Introduction

The ever-growing need for energy is pushing research towards the study and development of new energy storage and conversion tools with high efficiency such as Li-ion based batteries [1]. The request of stable low-cost components with a high energy-density is leading to the development of nanostructured metal oxides [2–4], because the nanostructuring allows a high specific capacity [5–13]. These considerations brought the development of a new variety of transition metal oxide based systems [14–24]. Within this context, iron oxide systems are convenient materials because of their low cost and environmental sustainability.

One of the important issues in Li-ion batteries is the chemical and thermal stability of the components. Fe_2_O_3_ presents a definite chemical phase (Fe^3+^) with a high chemical stability, while the mixed chemical state of Fe_3_O_4_ (Fe^2+/3+^) might induce instabilities during its use as electrode material. In the present work, we present a spectroscopic and morphologic characterization of Fe_2_O_3_ nanowires (NWs), which were produced by means of a hard template method [25] that allows for a good control over the size of the nanoparticles [26]. The characterization was carried out as a function of the annealing temperature in order to assess the thermal stability of the NWs and the temperatures, above which a chemical reduction of the Fe ions takes place. Thermogravimetry measurements distinctly show the mass reduction due to oxygen loss, and infrared transmittance and core-level photoemission measurements allow to follow the reduction process of the iron ions at different temperatures, showing the chemical reduction to Fe_3_O_4_ starting at moderate temperatures (above 440 K).

## Experimental

Thermogravimetry (TGA) measurements were performed by means of a Setaram Setsys Evolution 1200 apparatus, equipped with a mass spectrometer Pfeiffer Vacuum Quadstar QMS200. To identify all possible gaseous products, survey scans in the mass range between 1 and 100 amu were recorded. The TGA measurements were performed by heating in vacuum (approx. 10^−4^ mbar) at 0.5 K/min. Infrared spectra were collected by means of an Agilent Cary 660 spectrometer with a resolution of 1 cm^−1^ in the frequency range between 430 and 1100 cm^−1^. The spectra were the mean of at least 100 scans for each sample. The NW oxide powders were ground and mixed with dried KBr in a weight ratio of about 1:100. The mixed powders were pressed in a circular die in order to have self-standing pellets. The transmission of each sample was calculated as the ratio between the intensity transmitted by each pellet and the intensity transmitted by a pure potassium bromide pellet, produced in a similar way. Field-emission scanning electron microscopy (SEM) images have been taken at the Sapienza Nanotechnology and Nanoscience Laboratory (SSN-Lab), with a Zeiss Auriga 405 instrument (nominal resolution of 1.0 nm at maximum magnification, beam energy of 10 keV). The X-ray photoemission spectroscopy (XPS) measurements have been carried out at the Lotus laboratory at the “Università di Roma La Sapienza”, in an ultra-high vacuum (UHV) system with a base pressure of 1 × 10^−10^ mbar, un-monochromatized Al Kα photon source (*hν* = 1486.7 eV), hemispherical electron analyzer with a pass energy of 100 eV. The binding energy (BE) with respect to the Fermi level has been calibrated at the Au-4*f*_7/2_ core level (84.0 eV BE).

The iron oxide nanowires have been obtained by means of a hard template method. The hard template is mesoporous silica (SBA-15) synthesized through the sol–gel method. In order to embed the iron oxide nanowires, 0.01 M Fe(NO_3_)_3_·9H_2_O was dissolved in 50 mL of ethanol and added to 1 g of SBA-15. This solution was mixed at room temperature, dried at 310 K for 1 week, and the resulting powder was sintered at 820 K to promote the decomposition and dehydration of NO*_x_*. After etching, washing and filtering, we obtained the Fe_2_O_3_ nanowires. A detailed description of the production procedure has been reported elsewhere [25]. For the spectroscopic investigation, the nanowires were finally dispersed in ethanolic solution, deposited onto Si and Cu substrates, and dried in vacuum before analysis.

## Results and Discussion

**Figure 1:**
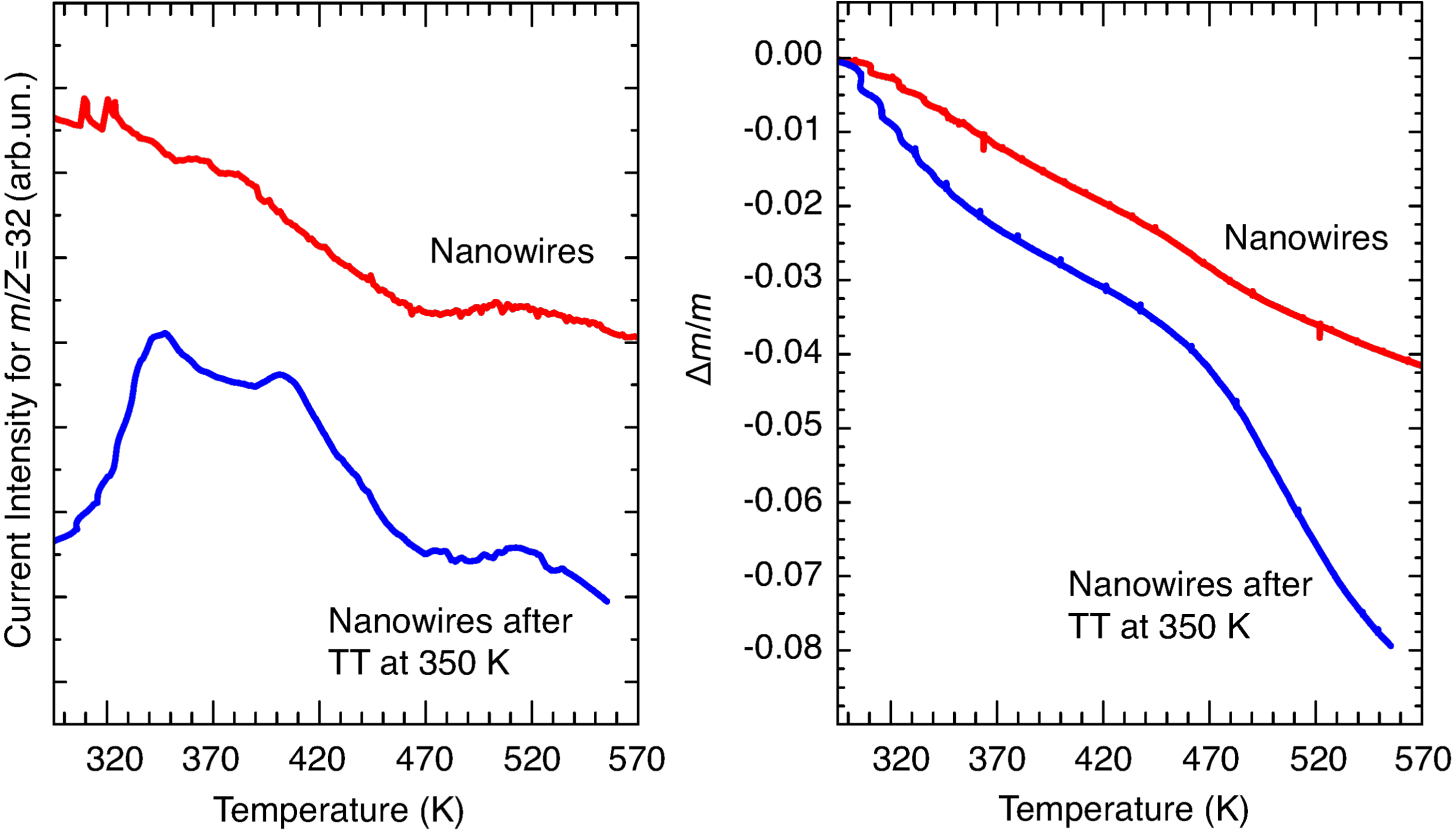
Temperature dependence of the signal of the mass spectrometer for *m*/*z* = 32 (left) and of the total mass variation Δ*m*/*m* (right), of the as-produced nanowire sample (red lines, sample 1) and of the nanowire specimen after a thermal treatment (TT) at 350 K for 24 h in vacuum (blue lines, sample 2).

TGA measurements were performed both on the as-produced nanowire sample (sample 1) and on a nanowire specimen heated in vacuum (p < 10^−4^ mbar) for 24 h at 350 K (sample 2), in order to clean the surface and to mimic the baking procedure that was carried out before the XPS measurements. In Figure 1 we report the mass variation of both samples and the correspondent signals detected by the mass spectrometer for *m*/*z* = 32 (oxygen molecule). Sample 1 displays a smooth, almost linear, loss of mass, which reaches a value of Δ*m*/*m* ≈ −0.03 around *T*_1_ = 470 K and increases further with higher temperatures. Correspondingly, the mass spectrometer detects a high value of the oxygen signal, which decreases with increasing temperatures. In the case of the nanowire specimen with a cleaned surface (sample 2), the mass variation is higher and reaches values of Δ*m*/*m* ≈ −0.04 around *T*_1_ = 470 K, and Δ*m*/*m* ≈ −0.08 around *T*_2_ = 560 K. The mass spectrum of Sample 2 displays a well evident oxygen peak below 470 K. These experiments suggest that oxygen loss from the nanowired samples takes place in any case below 470 K, even if its amount depends on the cleanliness of the surface. Indeed, the higher value of Δ*m*/*m* of the pre-heated sample (sample 2) suggests that the surface of the as-prepared nanowires can be covered by a layer acting as a barrier that prevents oxygen loss. However, these experiments are not conclusive about which iron oxide is obtained after the loss of O_2_. Therefore, we used infrared and XPS spectroscopy in order to identify the phase changes that are induced by the thermal treatment.

**Figure 2:**
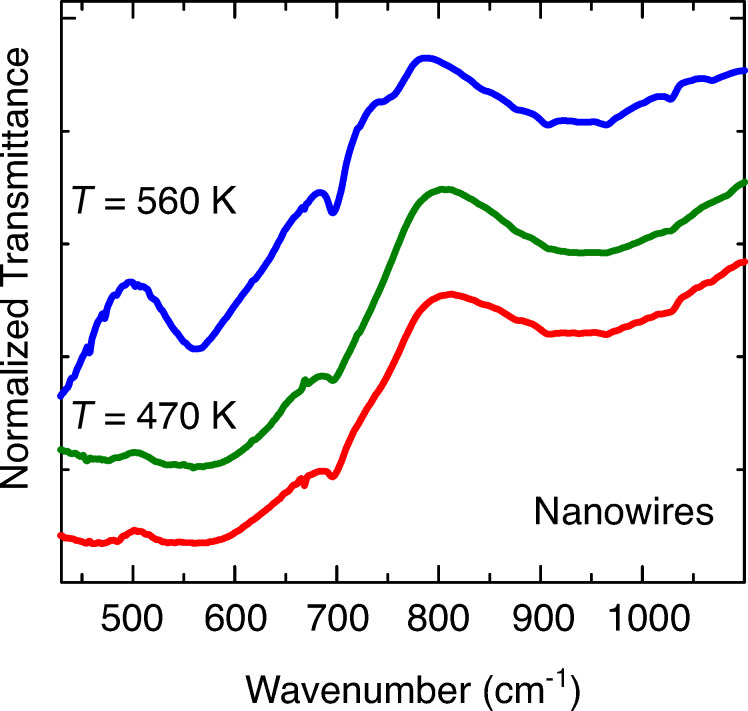
Infrared spectrum (normalized transmittance signal) of the nanowire sample (sample 2, red line), and of the specimen obtained from sample 2 after a thermal treatment at 470 K (sample 3, green line) and at 560 K (sample 4, blue line). In order to compare the transmission of different oxide powders, the transmittance spectra have been normalized. Data are vertically stacked, for the sake of clarity.

Infrared (IR) spectroscopy measurements were performed at room temperature (rt) on sample 2 and on two samples, which were obtained by heating sample 2 in vacuum (≈10^−4^ mbar) up to 470 K (sample 3) and up to 560 K (sample 4). The IR transmittance spectra of those samples are reported in Figure 2. Sample 2 shows an IR phonon spectrum that strongly resembles that of hematite, α-Fe_2_O_3_ [27], with a smooth transmittance between 500 and 650 cm^−1^ and the broad phonon band centered around 950 cm^−1^. However, we can observe a minimum of the transmittance around 700 cm^−1^, which is a fingerprint of maghemite (γ-Fe_2_O_3_) [27]. Thus, the clean sample 2 presents features that are typical of a mixture of α- and γ-Fe_2_O_3_. The infrared spectrum of the sample heated at 470 K (sample 3) is very similar to that of sample 2, while after the thermal treatment at 560 K (sample 4), the minimum around 700 cm^−1^ becomes deeper and the transmittance below 600 cm^−1^ decreases, which strongly resembles the infrared spectrum of magnetite, Fe_3_O_4_ [27].

The evolution of the infrared spectra with temperature indicates that in the pristine α-Fe_2_O_3_ material, there is a minor contribution of γ-Fe_2_O_3_, the concentration of which remains practically unchanged when the sample is heated to about 470 K, but increases significantly after a thermal treatment at *T*_2_ = 560 K. Moreover, at *T*_2_ a significant part of the sample is transformed into magnetite. We remark that the IR spectra are measured in transmission mode, so that they probe the whole thickness of the NW powders and are not limited to their surface. This issue is important to compare the IR conclusion with the results of the XPS core levels, with higher surface sensitivity.

**Figure 3:**
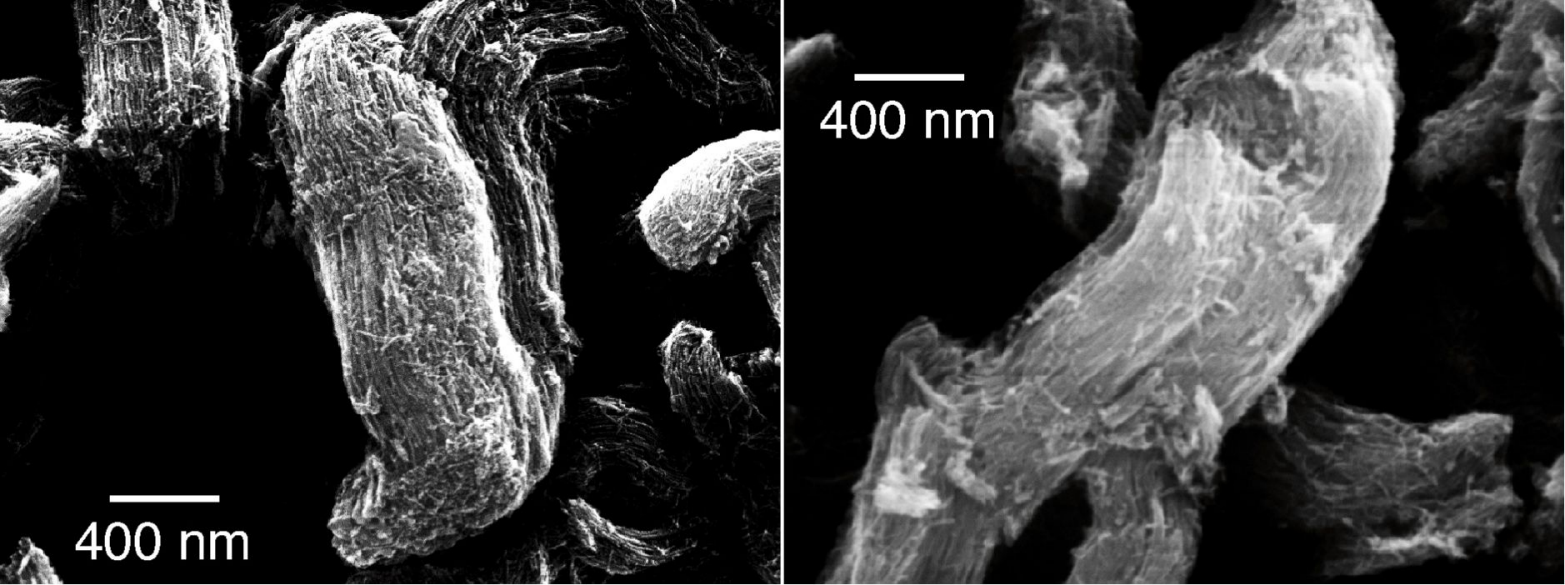
SEM images of the Fe_2_O_3_ nanowires deposited on a Si surface before (left) and after (right) thermal treatment at 650 K.

The iron oxide nanowires have been deposited onto a Si surface and imaged by SEM at rt before and after a thermal treatment at 650 K, to observe whether any morphology modification took place. the resulting images are shown in Figure 3. The NWs assemble in bundles that are a few hundreds of nanometers thick and several micrometers long. The individual NWs are visible within the bundles, as long parallel nanometer-thick rods. After the thermal treatment that changes the oxidation state of Fe in the NW, they do not change either shape or morphology in the bundled structure. Thus, the thermal treatment causes a chemical reduction, while not affecting the structure of the assembly, which renders the NWs a stable system for potential use in batteries, even after heating. We underline that heating at 650 K is by far a much higher temperature than what is to be expected in any device.

**Figure 4:**
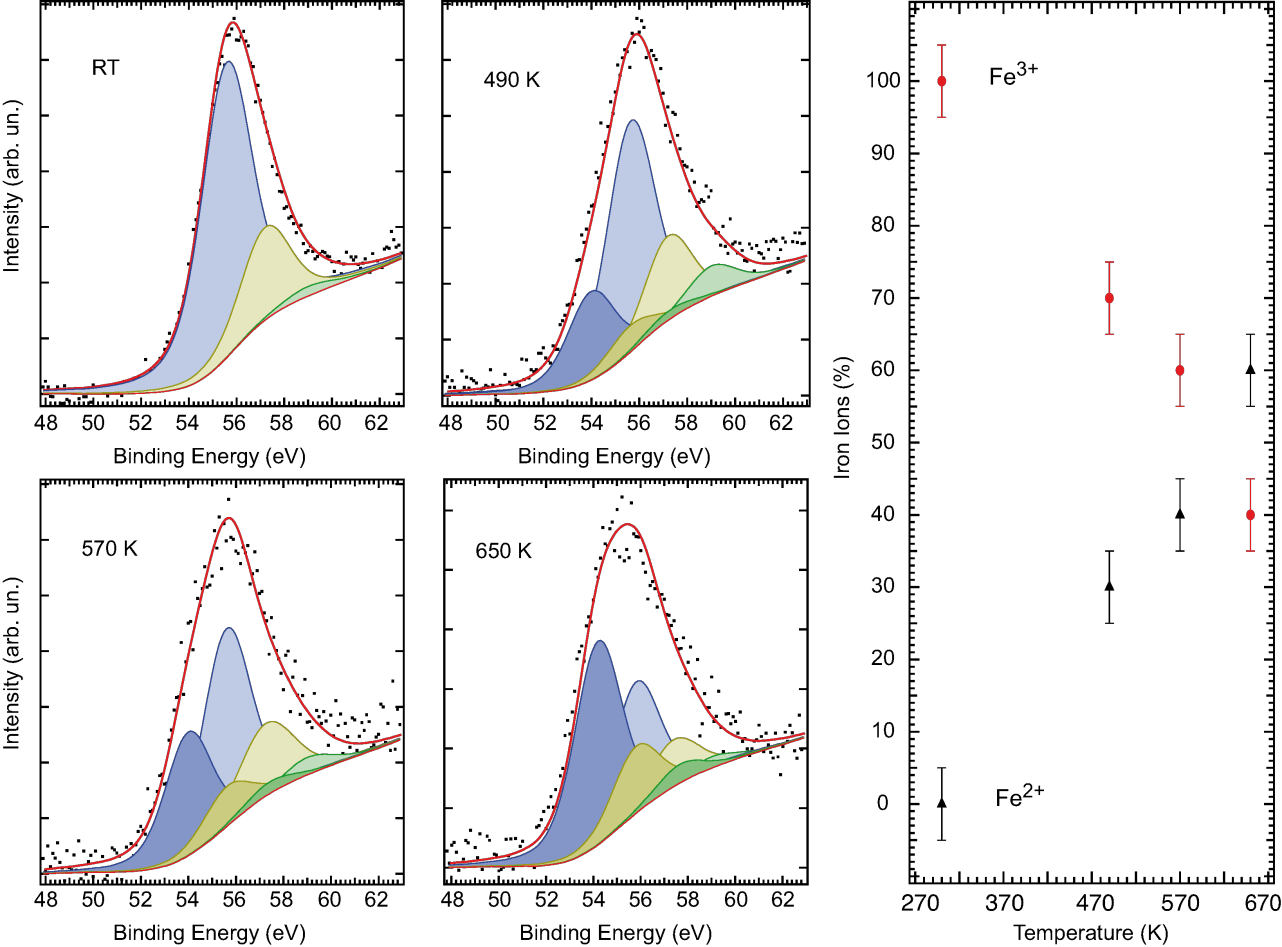
XPS of the Fe 3p core levels as a function of the annealing temperature. Left panels: XPS rough data (black dots), total fitting curves (red continuous lines), along with the deconvolution with contributions associated to the Fe^3+^ (light color curves) and Fe^2+^ (darker color curves) manifold components. Right panel: relative intensity estimations of the Fe^3+^ (red dots) and Fe^2+^ (black triangles) content in the NWs as a function of the temperature, as obtained from the fitting of the XPS experimental data (see text).

The oxidation state of the Fe atoms can be determined by an analysis of the Fe core levels [28–29]. We confirm the thermally induced reduction at moderate temperatures of the Fe ions in the NWs by the X-ray photoemission spectroscopy analysis of the Fe 3p core level. The XPS Fe 3p core-level data of the Fe_2_O_3_ NWs, taken at rt and after subsequent steps of thermal annealing, are shown in Figure 4. The Fe 3p signal of the clean Fe_2_O_3_ system at rt, which is roughly centered at 56 eV BE, presents the characteristic structure that is associated with multiple oxidation states [30–32].

We fit the experimental data with three Voigt (Lorentzian–Gaussian) functions with all peaks having the same Gaussian width (GW = 1.8 eV) and Lorentzian width (LW = 1.0 eV). The lineshape and BE of the Fe 3p core level confirm the Fe^3+^ oxidation state [32] of the Fe_2_O_3_ NWs. The Fe 3p XPS spectra that were taken after annealing the NWs at increasing temperatures present the emerging of a further manifold of peaks, at lower BE, the relative intensity of which grows as a function of temperature. This lower-BE manifold is associated to the Fe^2+^ oxidation state, and we fit it with three more Voigt functions, in analogy to the previous manifold. The evolution of the relative intensity of the Fe^3+^ and Fe^2+^ signal as evaluated from the fit, is shown in the right panel of Figure 4. Data analysis shows that already at 470 K, the reduction of iron ions has taken place, and finally a 60:40 ratio of Fe^2+^/Fe^3+^ is reached at 650 K. These spectroscopic data fully confirm the observed thermal-induced reduction of Fe_2_O_3_ to Fe_3_O_4_ at moderate temperatures.

The XPS measurements are more sensitive to the properties of the surface than the infrared spectroscopy measurements, in fact the electron mean free path of the photo-electrons is of the order of 1 nm in this energy range. Both experimental techniques indicate that at 470 K the sample has transformed into Fe_3_O_4_. However, while infrared measurements show an almost abrupt change from Fe_2_O_3_ to Fe_3_O_4_ between 470 K and 560 K, XPS measurements can sensitively detect the progressive change of the iron ion valence above room temperature. In particular, the appearance of divalent Fe ions is clearly visible above 440 K. In fact, XPS probes mainly the physical properties of the very surface. Therefore, the comparison between the data obtained by IR and XPS, strongly indicates that the reduction of iron oxide nanowires starts from their very surface and is completed in the bulk only around 560 K.

## Conclusion

We characterized the mass loss and spectroscopic change of Fe_2_O_3_ nanowires obtained through a hard template method [25], as a function of the annealing temperature, by means of thermogravimetry, IR and XPS spectroscopy. Heating the NWs induces an oxygen loss from the surface and a subsequent reduction of the Fe ions from a 3+ to a prevalent 2+ oxidation state at moderate temperatures (above 440 K). The reduction starts from the NW surface and progressively extends into the bulk, as determined by comparing the IR (bulk sensitive) and XPS (surface sensitive) techniques. Despite the chemical change, the NWs maintain the same shape and size, as imaged by SEM. The chemical reduction is clearly followed and quantified thanks to the thermogravimetry measurements and spectroscopic tools, and it assesses temperature limits for the operation of these nanowires in Li-ion based batteries, establishing the Fe_2_O_3_ nanowires as stable nanostructured elements for new advanced batteries.

## References

[1] Tarascon J-M, Armand M (2001). Nature.

[2] Poizot P, Laruelle S, Grugeon S, Dupont L, Tarascon J-M (2000). Nature.

[3] Taberna P L, Mitra S, Poizot P, Simon P, Tarascon J-M (2006). Nat Mater.

[4] Wu H B, Chen J S, Hng H H, Lou X W D (2012). Nanoscale.

[5] Larcher D, Masquelier C, Bonnin D, Chabre Y, Masson V, Leriche J-B, Tarascon J-M (2003). J Electrochem Soc.

[6] Chen Y X, He L H, Shang P J, Tang Q L, Liu Z Q, Liu H B, Zhou L P (2011). J Mater Sci Technol.

[7] Chou S-L, Wang J-Z, Chen Z-X, Liu H-K, Dou S-X (2011). Nanotechnology.

[8] Wang Z, Luan D, Madhavi S, Li C M, Lou X W D (2011). Chem Commun.

[9] Wang Z, Zhou L, Lou X W D (2012). Adv Mater.

[10] Zhang Q, Shi Z, Deng Y, Zheng J, Liu G, Chen G (2012). J Power Sources.

[11] Koo B, Xiong H, Slater M D, Prakapenka V B, Balasubramanian M, Podsiadlo P, Johnson C S, Rajh T, Shevchenko E V (2012). Nano Lett.

[12] Ding Y, Li J, Zhao Y, Guan L (2012). Mater Lett.

[13] Zhang J, Huang T, Liu Z, Yu A (2013). Electrochem Commun.

[14] Zou Y, Wang Y (2011). ACS Nano.

[15] Li X, Lei Y, Li X, Song S, Wang C, Zhang H (2011). Solid State Sci.

[16] Chen D, Ji G, Ma Y, Lee J Y, Lu J (2011). ACS Appl Mater Interfaces.

[17] Qu Q, Yang S, Feng X (2011). Adv Mater.

[18] Hsieh C-T, Lin J-Y, Mo C-Y (2011). Electrochim Acta.

[19] Lee S-H, Sridhar V, Jung J-H, Karthikeyan K, Lee Y-S, Mukherjee R, Koratkar N, Oh I-K (2013). ACS Nano.

[20] Guo S, Zhang G, Guo Y, Yu J C (2013). Carbon.

[21] Wang G, Wang H, Cai S, Bai J, Ren Z, Bai J (2013). J Power Sources.

[22] Jin B, Liu A-H, Liu G-Y, Yang Z-Z, Zhong X-B, Ma X-Z, Yang M, Wang H-Y (2013). Electrochim Acta.

[23] Prakash R, Fanselau K, Ren S, Mandal T K, Kübel C, Hahn H, Fichtner M (2013). Beilstein J Nanotechnol.

[24] Zhao H, Pan L, Xing S, Luo J, Xu J (2013). J Power Sources.

[25] Hong I, Angelucci M, Verrelli R, Betti M G, Panero S, Croce F, Mariani C, Scrosati B, Hassoun J (2014). J Power Sources.

[26] Reddy M V, Yu T, Sow C H, Shen Z X, Lim C T, Subba Rao G V, Chowdari B V R (2007). Adv Funct Mater.

[27] Cornell R M, Schwertmann U (2003). The Iron Oxides.

[28] Chambers S A, Thevuthasan S, Joyce S A (2000). Surf Sci.

[29] Yamashita T, Hayes P (2008). Appl Surf Sci.

[30] Yang D-Q, Sacher E (2009). J Phys Chem C.

[31] Mills P, Sullivan J L (1983). J Phys D: Appl Phys.

[32] Zimmermann R, Steiner P, Claessen R, Reinert F, Hüfner S, Blaha P, Dufek P (1999). J Phys: Condens Matter.

